# Supplementary material for: Light-triggered and phosphorylation-dependent 14-3-3 association with NON-PHOTOTROPIC HYPOCOTYL 3 is required for hypocotyl phototropism
Source: Nat Commun. 2021 Oct 21;12:6128. doi: 10.1038/s41467-021-26332-6 (PMC8531446; doi:10.1038/s41467-021-26332-6)
Supplement: Supplementary file 1 — Supplementary Information [file 41467_2021_26332_MOESM1_ESM.pdf]

SUPPLEMENTARY INFORMATION

Light-triggered and phosphorylation-dependent 14-3-3 association with NON-PHOTOTROPIC HYPOCOTYL 3 is required for hypocotyl phototropism

Lea Reuter[1], Tanja Schmidt[1], Prabha Manishankar[1], Christian Throm[1], Jutta Keicher[1], Andrea Bock[1], Irina Droste-Borel[2], Claudia Oecking[1]*

[1]Center for Plant Molecular Biology (ZMBP), Plant Physiology, University of Tübingen, Tübingen, Germany.
[2]Proteome Center Tübingen, University of Tübingen, Tübingen, Germany.

These authors contributed equally: Lea Reuter, Tanja Schmidt
* Corresponding author, email: claudia.oecking@zmbp.uni-tuebingen.de

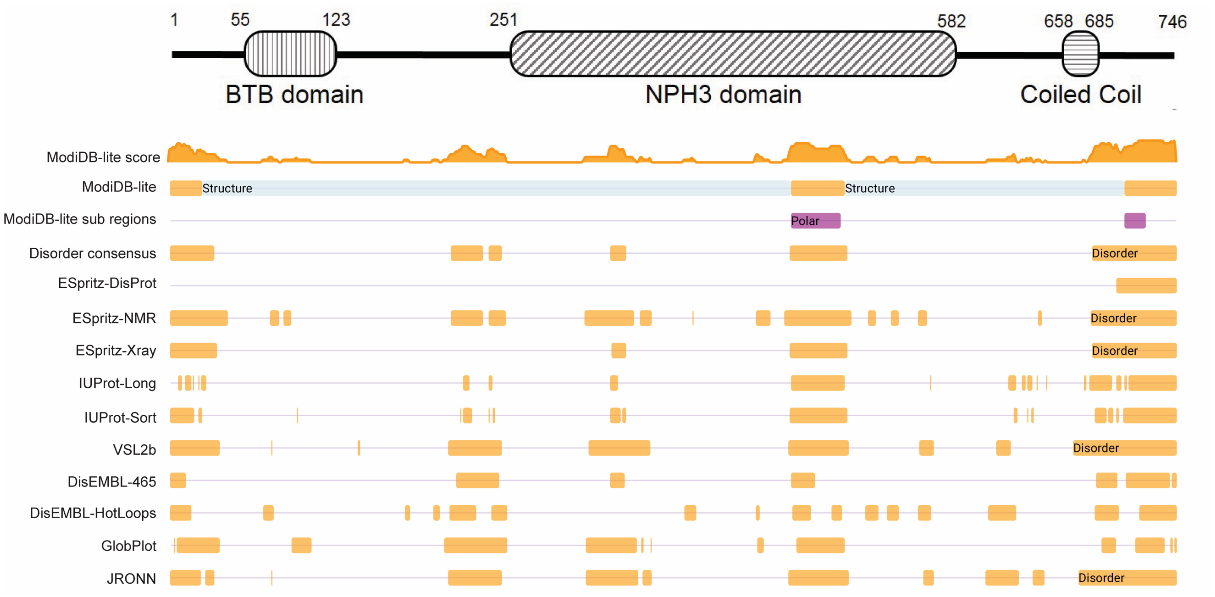

Supplementary Fig. 1. **Domain structure of NPH3.** Domain structure of NPH3 and MobiDB plot (https://mobidb.org/) of intrinsically disordered regions in NPH3. BTB domain, bric-a-brac, tramtrack and broad complex domain.

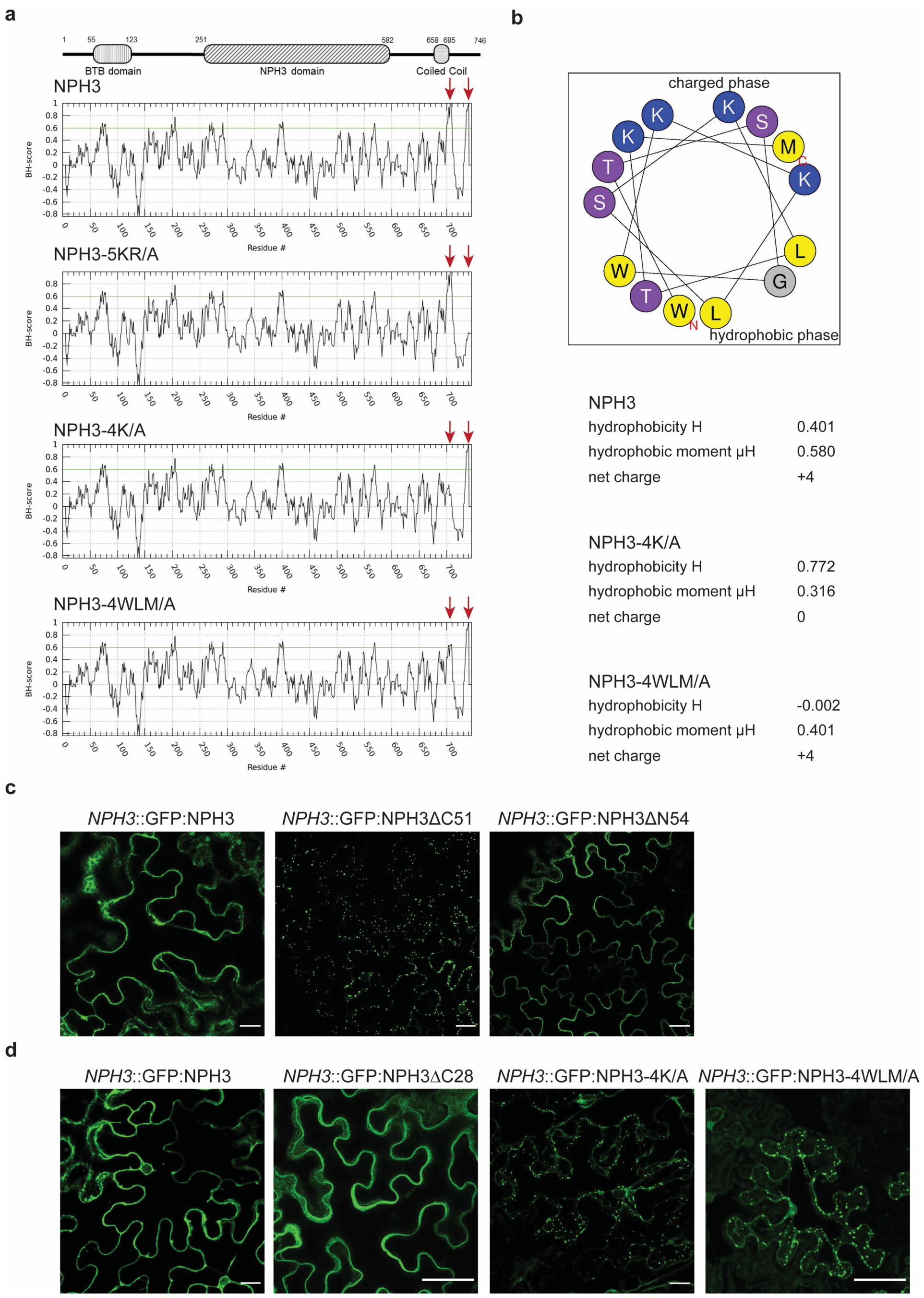

Supplementary Fig. 2. **An amphipathic helix within the C-terminal domain is required for plasma membrane localization of NPH3. a** BH score profiles (window size 11) of NPH3 and mutant variants (see Fig. 2a). Putative BH-domains are indicated by red arrows. **b** Helical wheel projection showing amphipathy of the predicted helix (residues 700-713) within the C-terminal domain of NPH3. Overall helix hydrophobicity (H) and the hydrophobic moment (µH) are given for NPH3 and mutant variants, respectively. **c** Representative confocal microscopy images of leaf epidermal cells from dark-adapted *N. benthamiana* transiently expressing GFP:NPH3ΔC51 or GFP:NPH3ΔN54. Expression was driven by the *NPH3* promoter. GFP:NPH3 is shown as control. Scale bars, 25 µm. **d** Representative confocal microscopy images of leaf epidermal cells from dark-adapted *N. benthamiana* transiently expressing GFP:NPH3 variants characterized by mutations within the two putative BH motifs (see Fig. 2a). Expression was driven by the *NPH3* promoter. Z-stack projections are shown. GFP:NPH3 is shown as control. Scale bars, 25 µm. Experiments in **c** and **d** were performed at least three times with similar results.

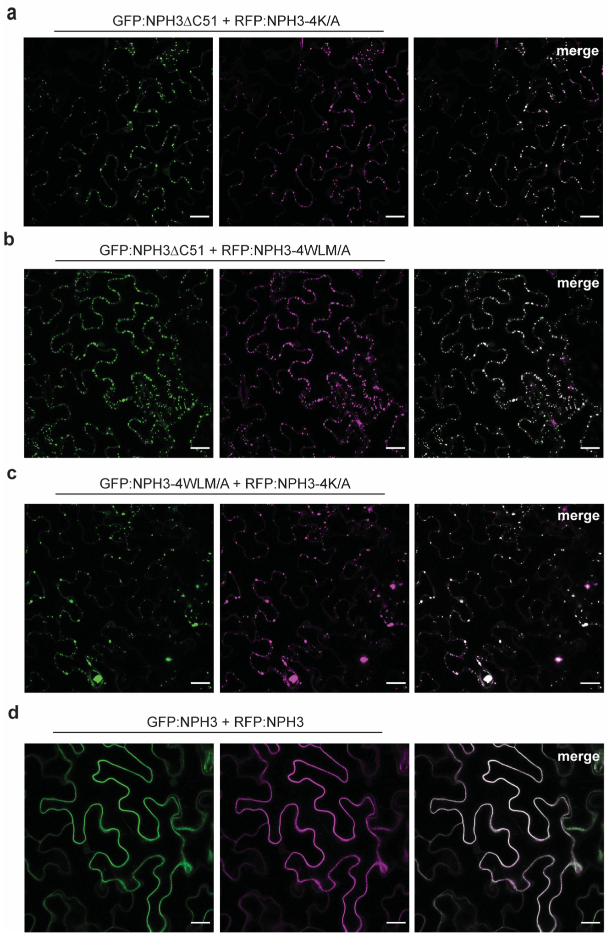

Supplementary Fig. 3. **Co-localization of NPH3 mutants constitutively localizing to condensates. a, b, c, d** Representative confocal microscopy images of leaf epidermal cells from dark-adapted *N. benthamiana* transiently co-expressing fluorophore-tagged NPH3 variants that constitutively localize to condensates (**a**, **b**, **c**). Expression was driven by the 35S promoter. Co-expression of fluorophore-tagged NPH3 is shown as control (**d**). Scale bars, 25 μm. All co-expression analyses were performed at least three times with similar results.

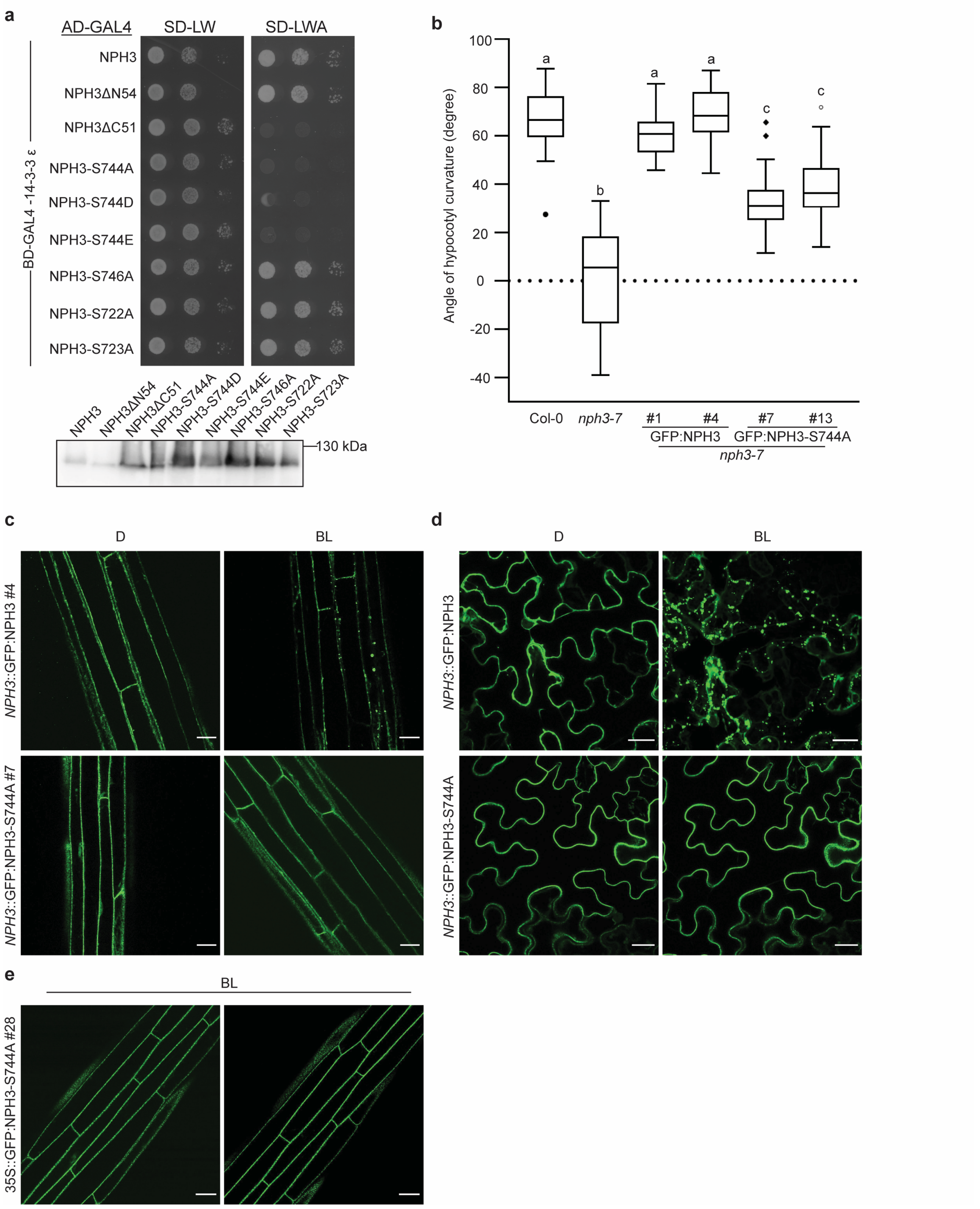

Supplementary Fig. 4. **14-3-3 binding is required for NPH3 function and its BL-induced dissociation from the plasma membrane. a** Yeast two-hybrid interaction analysis of the Arabidopsis 14-3-3 isoform epsilon with NPH3 variants (upper panel). Expression of the diverse NPH3 fusion proteins was confirmed by anti-HA immunoblot of yeast extracts (lower panel). AD, activating domain, BD, binding domain. **b** Quantification of hypocotyl phototropism (mean $\pm$ SD) in etiolated Arabidopsis *nph3-7* seedlings expressing either GFP:NPH3 or GFP:NPH3-S744A. Expression was driven by the *NPH3* promoter. Seedlings were exposed for 24 h to unilateral blue light (BL) (1 μmol m^{-2} sec^{-1}) ($n \geq 30$ seedlings per experiment, one representative experiment of three replicates is presented). One-way ANOVA with Tukey's *post hoc* test is shown, different letters mark statistically significant differences ($P<0.05$), same letters mark statistically non-significant differences. Centre line: median, bounds of box: minima and maxima (25th and the 75th percentiles), whiskers: 1.5 * IQR (IQR: the interquartile range between the 25th and the 75th percentile). Exact P values for all experiments are provided in the source data file. **c** Representative confocal microscopy images of hypocotyl cells from 3-days old transgenic etiolated Arabidopsis *nph3-7* seedlings

shown in **b**. The plants were either kept in darkness (D) or treated with BL (1 μmol m^{-2} sec^{-1}) for 40 min. Scale bars, 25 μm. **d** Representative confocal microscopy images of leaf epidermal cells from *N. benthamiana* expressing either GFP:NPH3 or GFP:NPH3-S744A. Expression was driven by the *NPH3* promoter. Dark-adapted tobacco plants were either kept in D or treated with BL (approx. 11 min GFP-laser). Z-stack projection of BL-treated GFP:NPH3 is shown. Scale bar, 25 μm. **e** Representative confocal microscopy images of hypocotyl cells from transgenic etiolated Arabidopsis *nph3-7* seedlings expressing GFP:NPH3-S744A. Expression was driven by the 35S promoter. Etiolated seedlings were treated with BL (1 μmol m^{-2} sec^{-1}) for 40 min. Scale bars, 25 μm. All experiments were performed at least three times with similar results.

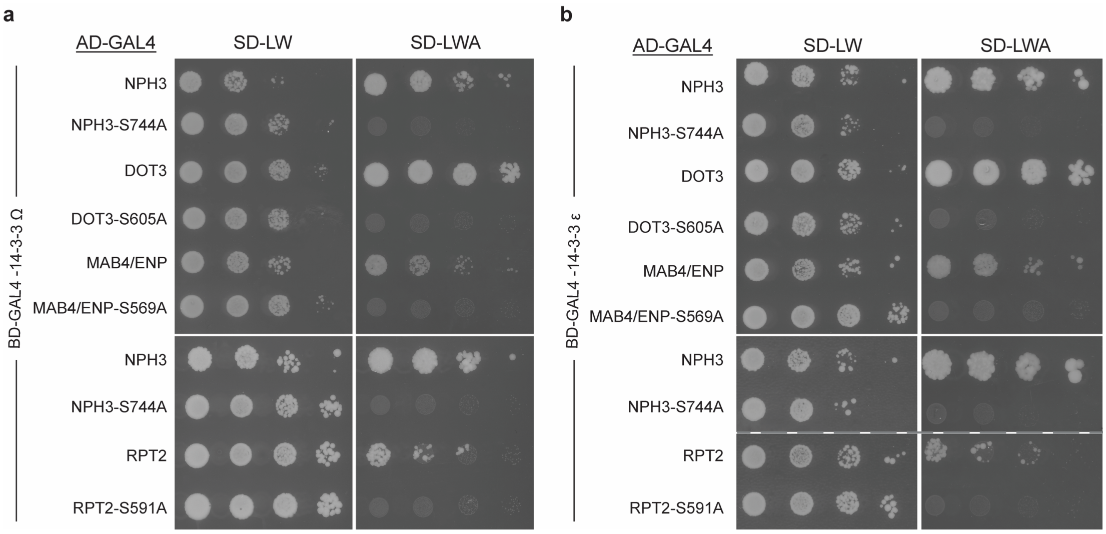

Supplementary Fig. 5. **Several NRL protein family members interact with 14-3-3 proteins via a C-terminal binding motif.** Yeast two-hybrid interaction analysis of the Arabidopsis 14-3-3 isoform omega (**a**) and epsilon (**b**) with various NRL wild type and mutant variants (exchange of the third last residue (serine (S) to alanine (A)), respectively). Yeast growth was recorded after 4 days (upper panel) or 6 days (lower panel). The analyses were performed at least three times with similar results.

Supplementary Table 1. **Blue-light dependent 14-3-3 epsilon interactors in etiolated Arabidopsis seedlings.** Analysis of 14-3-3 epsilon-GFP immunoprecipitates via mass spectrometry (MS) based on two biological replicates. This table lists only known 14-3-3 clients in addition to NPH3.

AGI code	gene name	description	Mol. weight (kDa)	peptides R1		Sequence coverage R1 (%)		Intensity R1		Normalized intensity R1		Intensity (BL/dark) R1
				dark	BL	dark	BL	dark	BL	dark	BL	
AT1G22300	GRF10	14-3-3-like protein GF14 epsilon	28.90	32	30	79.5	79.5	3.23E+11	3.74E+11	3.00E+11	3.00E+11	1.00
AT1G35580	CINV1	Alkaline/neutral invertase CINV1	62.83	35	32	64.2	64.2	3.50E+10	2.98E+10	3.25E+11	2.39E+10	0.74
AT2G18960	AHA1	ATPase 1, plasma membrane-type	104.22	31	36	36.4	43.4	2.97E+09	4.41E+09	2.76E+10	3.54E+09	1.28
AT4G30190	AHA2	ATPase 2, plasma membrane-type	104.40	33	37	39.9	42.6	4.02E+08	3.81E+08	3.73E+09	3.05E+08	0.82
AT5G64330	NPH3	Non-phototropic hypocotyl 3	81.87	5	22	7.4	39.0	5.35E+07	1.15E+09	4.97E+08	9.19E+08	18.5
AT1G09570	PhyA	Phytochrome A	125.02	1	1	0.9	1.2	4.66E+06	5.50E+06	4.32E+06	4.41E+06	1.02
AT5G11110	SPS1	Sucrose-phosphate synthase 1	117.32	13	18	15.6	22.7	2.46E+08	5.19E+08	2.29E+08	4.17E+08	1.82
AT5G03280	EIN2	Ethylene-insensitive protein 2	140.95	6	9	6.6	9.0	8.10E+07	1.36E+08	7.52E+07	1.09E+08	1.45
AGI code	gene name	description	Mol. weight (kDa)	peptides R2		Sequence coverage R2 (%)		Intensity R2		Normalized intensity R2		Intensity (BL/dark) R2
				dark	BL	dark	BL	dark	BL	dark	BL	
AT1G22300	GRF10	14-3-3-like protein GF14 epsilon	28.90	33	33	76.4	76.4	3.34E+11	3.99E+11	3.00E+11	3.00E+11	1.00
AT1G35580	CINV1	Alkaline/neutral invertase CINV1	62.83	31	30	61.2	57.0	2.88E+10	4.40E+10	2.59E+10	3.30E+10	1.27
AT2G18960	AHA1	ATPase 1, plasma membrane-type	104.22	33	30	41.5	36.1	2.77E+08	2.96E+08	2.48E+08	2.22E+08	0.89
AT4G30190	AHA2	ATPase 2, plasma membrane-type	104.40	34	31	38.9	36.0	3.32E+09	3.48E+09	2.98E+09	2.61E+09	0.87
AT5G64330	NPH3	Non-phototropic hypocotyl 3	81.87	1	14	1.5	24.4	1.13E+06	5.03E+08	1.00E+06	3.77E+08	377.0
AT1G09570	PhyA	Phytochrome A	125.02	4	1	5.1	1.6	2.15E+07	4.28E+06	1.93E+07	3.21E+06	0.17
AT5G11110	SPS1	Sucrose-phosphate synthase 1	117.32	8	9	12.8	14.4	1.36E+08	2.38E+08	1.22E+08	1.78E+08	1.46
AT5G03280	EIN2	Ethylene-insensitive protein 2	140.95	5	3	5.1	2.7	4.83E+07	4.64E+07	4.34E+07	3.50E+07	0.81

Supplementary Table 2. **List of primers used in this study**

pGEM-T Easy/ yeast & bacterial expression vectors/ mutagenesis	
NPH3_SmaI_F	TATcccgggCATGTGGGAATCTGAGAGCGAC
NPH3ΔN53_Sma_F	TATcccgggCGATCTTCTGGTTAAGATCGGC
NPH3-C51_EcoRI_F	TATgaattcTCTTCTTCGGCTTGGACCAGC
NPH3_SalI_R	TATgtcgacTCATGAAATTGAGTTCCT
NPH3ΔC51_SalI_R	TATgtcgacCTATGGCGTGTTCTTCACTTTCCC
NPH3_S743A_SalI_R	TATgtcgacTCATGAAATTGcGTTCCTCCATCGTCT
NPH3_S743D_SalI_R	TATgtcgacTCATGAAATgtcGTTCCTCCATCGTCTTGGTTTC
NPH3_S743E_SalI_R	TATgtcgacTCATGAAATTtcGTTCCTCCATCGTCTTGGTTTC
NPH3_S745A_SalI_R	TATgtcgacTCATGcAATTGAGTTCCTCCATCGTCT
NPH3_3KR/A_SalI_R	TATgtcgacTCATGAAATTGAGTTCgcCCATgcTCTTGGTTTCgcGGGGGGTGGATGATC
NPH3_5KR/A_SalI_R	TATgtcgacTCATGAAATTGAGTTCgcCCATgcTgcTGGTgcCgcGGGGGGTGGATGATC
NPH3_ 4K/A_F	GCTTGGACCAGCGGTTGGgcGgcGCTAAGTgcACTGACTgcGATGAGTGGACAGGAGAG
NPH3_ 4K/A_R	CTCTCCTGTCCACTCATCgcAGTCAGTgcACTTAGCgcCgcCCAACCGCTGGTCCAAGC
NPH3_4WLM/A_F	TCGGCTTGGACCAGCGGTgcGAAGAAGgcAAGTAAAgcGACTAAGgcGAGTGGACAGGAGAGCCAT
NPH3_4WLM/A_R	ATGGCTCTCCTGTCCACTCgcCTTAGTCgcTTTACTTgcCTTCTTCgcACCGCTGGTCCAAGCCGA
NPH3_S721A_F	CAGGAGAGCCATGACATAGCCTCTGGAGGAGAACAAGCT
NPH3_S721A_R	AGCTTGTTCTCCTCCAGAGGCTATGTCATGGCTCTCCTG
NPH3_S722A_F	GAGAGCCATGACATATCCGCTGGAGGAGAACAAGCTGGT
NPH3_S722A_R	ACCAGCTTGTTCTCCTCCAGCGGATATGTCATGGCTCTC
14-3-3omega_BamHI_F	TATggatccATGGCGTCTGGGCGTGAAGAG
14-3-3omega_EcoRI_F	TATgaattcATGGCGTCTGGGCGTGAAGAG
14-3-3omega_SalI_R	TATgtcgacTCACTGCTGTTCCTCGGT
GATEWAY	
NPH3_attB1_F	AAAAAGCAGGCTTAATGTGGGAATCTGAGAGCGAC
NPH3ΔN53_attB1_F	AAAAAGCAGGCTTAATGGATCTTCTGGTTAAGATCGGC
NPH3_attB2_R	AGAAAGCTGGGTGTCATGAAATTGAGTTCCTCCA
NPH3_S743A_attB2_R	AGAAAGCTGGGTGTCATGAAATTGcGTTCCTCCATCGTCT
NPH3_S743D_attB2_R	AGAAAGCTGGGTGTCATGAAATgtcGTTCCTCCATCGTCT
NPH3_5KR/A_attB2_R	AGAAAGCTGGGTGTCATGAAATTGAGTTCgcCCATgcTgcTGG
NPH3ΔC28_attB2_R	AGAAAGCTGGGTGTCAGCTCTCCTGTCCACTCATCTT
NPH3ΔC51_attB2_R	AGAAAGCTGGGTGCTATGGCGTGTTCTTCACTTTCCC
NPH3_attB4_R	GAAAAGTTGGGTGTCATGAAATTGAGTTCCTCCA
NPH3_S743A_attB4_R	GAAAAGTTGGGTGTCATGAAATTGCGTTCCTCCATCGTCT

14-3-3omega_attB3_F	ataataaagttgtaATGGCGTCTGGGCGT
14-3-3omega_attB2_R	agaaagctgggtgCTGCTGTTCCTCGGT
attB1 adapter	GGGGACAAGTTTGTACAAAAAAGCAGGCT
attB2 adapter	GGGGACCACTTTGTACAAGAAAGCTGGGT
attB3 adapter	GGGGACAACTTTGTATAATAAAGTTG
attB4 adapter	GGGGACAACTTTGTATAGAAAAGTTGGGT
GOLDEN GATE	
NPH3prom_A-B_F	AACAggtctcAGCGGAAACCCCACATTAATCAGACAGAATC
NPH3prom_A-B_R	AACAggtctcACAGAACACAAGTTAACACTCTCTGTAGTTG
NPH3_C-D_F	AACAggtctcACACCATGTGGGAATCTGAGAGCGAC
NPH3ΔN53_C-D_F	AACAggtctcACACCATGGATCTTCTGGTTAAGATCGGC
NPH3_C-D_R	AACAggtctcACCTTTCATGAAATTGAGTTCCTCCA
NPH3_S743A_C-D_R	AACAggtctcACCTTTCATGAAATTGCGTTCCTCCATCGTCT
NPH3ΔC28_C-D_R	AACAggtctcACCTTTCAGCTCTCCTGTCCACTCATCTT
NPH3ΔC51_C-D_R	AACAggtctcACCTTTCATGGCGTGTTCTTCACTTTCCC
