# Supplementary material for: Light-triggered and phosphorylation-dependent 14-3-3 association with NON-PHOTOTROPIC HYPOCOTYL 3 is required for hypocotyl phototropism
Source: Nat Commun. 2021 Oct 21;12:6128. doi: 10.1038/s41467-021-26332-6 (PMC8531446; doi:10.1038/s41467-021-26332-6)
Supplement: Supplementary file 3 — Description of Additional Supplementary Files [file 41467_2021_26332_MOESM3_ESM.pdf]

Description of Additional Supplementary Files

File name: Supplementary Movie 1

Description: Dynamic BL-induced changes in the subcellular localization of 35S::GFP:NPH3 in hypocotyl cells of stably transformed Arabidopsis *nph3-7*.

File name: Supplementary Movie 2

Description: Subcellular localization of 35S::GFP:NPH3-S744A in hypocotyl cells of stably transformed Arabidopsis *nph3-7* upon BL-irradiation.

File name: Supplementary Movie 3

Description: Dynamic BL-induced changes in the subcellular localization of 35S::RFP:NPH3 transiently expressed in *N. benthamiana* leaves.

File name: Supplementary Movie 4

Description: Subcellular localization of 35S::RFP:NPH3-S744A in transiently transformed *N. benthamiana* leaves upon BL-irradiation.

File name: Supplementary Movie 5

Description: Subcellular localization of 35S::GFP:NPH3-4K/A in hypocotyl cells of stably transformed Arabidopsis *nph3-7* upon BL-irradiation.

File name: Supplementary Movie 6

Description: Subcellular localization of 35S::GFP:NPH3DC51 in hypocotyl cells of stably transformed Arabidopsis *nph3-7* upon BL-irradiation.

File name: Supplementary Movie 7

Description: Subcellular localization of 35S::GFP:NPH3DC28 in hypocotyl cells of stably transformed Arabidopsis *nph3-7* upon BL-irradiation.

File name: Supplementary Movie 8

Description: Dynamic BL-induced changes in the subcellular localization of 35S::RFP:NPH3DN54 transiently expressed in *N. benthamiana* leaves.

File name: Supplementary Movie 9

Description: Dynamic BL-induced changes in the subcellular localization of 35S::GFP:NPH3DN54 in hypocotyl cells of stably transformed Arabidopsis *nph3-7*.
