# Supplementary material for: Light-triggered and phosphorylation-dependent 14-3-3 association with NON-PHOTOTROPIC HYPOCOTYL 3 is required for hypocotyl phototropism
Source: Nat Commun. 2021 Oct 21;12:6128. doi: 10.1038/s41467-021-26332-6 (PMC8531446; doi:10.1038/s41467-021-26332-6)
Supplement: Supplementary file 13 — Reporting Summary [file 41467_2021_26332_MOESM13_ESM.pdf]

nature portfolio

Corresponding author(s): Claudia Oecking

Last updated by author(s): Sep 16, 2021

Reporting Summary

Nature Portfolio wishes to improve the reproducibility of the work that we publish. This form provides structure for consistency and transparency in reporting. For further information on Nature Portfolio policies, see our Editorial Policies and the Editorial Policy Checklist.

Statistics

For all statistical analyses, confirm that the following items are present in the figure legend, table legend, main text, or Methods section.

n/a	Confirmed	
☐	☒	The exact sample size (n) for each experimental group/condition, given as a discrete number and unit of measurement
☐	☒	A statement on whether measurements were taken from distinct samples or whether the same sample was measured repeatedly
☐	☒	The statistical test(s) used AND whether they are one- or two-sided<br>*Only common tests should be described solely by name; describe more complex techniques in the Methods section.*
☒	☐	A description of all covariates tested
☒	☐	A description of any assumptions or corrections, such as tests of normality and adjustment for multiple comparisons
☐	☒	A full description of the statistical parameters including central tendency (e.g. means) or other basic estimates (e.g. regression coefficient) AND variation (e.g. standard deviation) or associated estimates of uncertainty (e.g. confidence intervals)
☐	☒	For null hypothesis testing, the test statistic (e.g. F, t, r) with confidence intervals, effect sizes, degrees of freedom and P value noted<br>*Give P values as exact values whenever suitable.*
☒	☐	For Bayesian analysis, information on the choice of priors and Markov chain Monte Carlo settings
☒	☐	For hierarchical and complex designs, identification of the appropriate level for tests and full reporting of outcomes
☒	☐	Estimates of effect sizes (e.g. Cohen's d, Pearson's r), indicating how they were calculated

Our web collection on statistics for biologists contains articles on many of the points above.

Software and code

Policy information about availability of computer code

Data collection	Leica Application Suite X (3.5.7.23225), AMERSHAM ImageQuant 800, EPSON Scanner- EPSON PERFECTION V700
Data analysis	Single-cell time-lapse imaging: done according to protocol published by Zavaliev and Epel 2014 (https://link.springer.com/protocol/10.1007%2F978-1-4939-1523-1_7#Sec7) using ImageJ software (https://imagej.nih.gov/ij/) , parameters: Rolling ball radius:10; Filters, Mean:1; Bernsen radius:10; size range of ROI objects:3-400 pixel2; circularity range:0,5-1<br>Confocal images: LAS X (versions 3.5.7.23225 and/or 3.3.0.16799), Photoshop (version CS5, https://www.photoshop.com)<br>Immunoblots: ImageJ (https://imagej.nih.gov/ij/), Photoshop (version CS5, https://www.photoshop.com)<br>Angle of hypocotyl curvature: Image J (https://imagej.nih.gov/ij/)<br>Statistical analysis: MS Office Excel (https://www.microsoft.com/microsoft-365/excel) for Single-cell time-lapse imaging and IP-MS, Graphpad Prism (Version 9.2, https://www.graphpad.com) for Phototropism<br>Processing of MS data: MaxQuant software (https://www.maxquant.org, Version 1.5.2.8. with integrated Andromeda Peptid search engine). The spectra were searched against an Arabidopsis thaliana database (UP000006548_3702_complete_2017-12-20.fasta)<br>Webtools used<br>BH motif search: https://hpcwebapps.cit.nih.gov/bhsearch/<br>Analysis of physicochemical properties of a helix: https://heliquest.ipmc.cnrs.fr

For manuscripts utilizing custom algorithms or software that are central to the research but not yet described in published literature, software must be made available to editors and reviewers. We strongly encourage code deposition in a community repository (e.g. GitHub). See the Nature Portfolio guidelines for submitting code & software for further information.

Data

Policy information about availability of data

All manuscripts must include a data availability statement. This statement should provide the following information, where applicable:
- Accession codes, unique identifiers, or web links for publicly available datasets
- A description of any restrictions on data availability
- For clinical datasets or third party data, please ensure that the statement adheres to our policy

All data are available within this Article and its Supplementary Information.

Field-specific reporting

Please select the one below that is the best fit for your research. If you are not sure, read the appropriate sections before making your selection.

☒ Life sciences ☐ Behavioural & social sciences ☐ Ecological, evolutionary & environmental sciences

For a reference copy of the document with all sections, see nature.com/documents/nr-reporting-summary-flat.pdf

Life sciences study design

All studies must disclose on these points even when the disclosure is negative.

Sample size	Single-cell time-lapse imaging: Zavaliev et al. 2020, repeated 5 times<br>Confocal imaging: repeated at least 3 times (N. benthamiana infiltration: 3 technical replicates per biological replicate experiment)<br>Immunoblots: repeated at least 3 times<br>Phototropism: ≥30 seedlings for each genotype in each biological replicate (3 biological replicates in total)
Data exclusions	No data were exluded from the analyses provided.
Replication	Reproducibility of data was tested by multiple repetitions of the experiments described. All experiments were conducted at least 3 times on different days using biological materials produced independently (biological replicates). Statistical evaluation was applied to all data sets obtained and is mentioned in figure legends when applicable. All attempts at replication were successful.
Randomization	Allocation of test plants used in our study was random. There was no targeted selection of individual plants for specific treatments.
Blinding	Blinding was not used in our study as it does not include clinical trials. In plant biology blinded/double-blinded studies are uncommon.

Reporting for specific materials, systems and methods

We require information from authors about some types of materials, experimental systems and methods used in many studies. Here, indicate whether each material, system or method listed is relevant to your study. If you are not sure if a list item applies to your research, read the appropriate section before selecting a response.

Materials & experimental systems

n/a	Involved in the study	
☐	☒	Antibodies
☒	☐	Eukaryotic cell lines
☒	☐	Palaeontology and archaeology
☒	☐	Animals and other organisms
☒	☐	Human research participants
☒	☐	Clinical data
☒	☐	Dual use research of concern

Methods

n/a	Involved in the study	
☒	☐	ChIP-seq
☒	☐	Flow cytometry
☒	☐	MRI-based neuroimaging

Antibodies

Antibodies used	Primary Antibodies Used:<br>• anti-HA high affinity: Roche, Cat.-No. 11867423001, Lot unknown, Dilution: 1:2,000<br>• anti-GST: Cytiva, Cat.-No. 27457701, Lot: 5205496, Dilution: 1:2,000<br>• anti-GFP: Thermo Scientific, Cat.-No. A-11122, Lot: 2180255, Dilution: 1:1,000<br>• anti-RFP: Chromotek, Cat.-No. 5F8-100, Lot:90228002AB-10, Dilution: 1:1,000<br>• anti-pS744 (Dilution 1:500), anti-NPH3 (Dilution 1:1,000): Eurogentec, custom made, antigen: NH2-PPRKPRRWRN-S(PO3H2)-IS-COOH, affinity-purifications against the non-phosphorylated and phosphorylated peptide<br>• anti-RGS His: Qiagen, Cat.-No. 34650, Lot: 163033250, Dilution:1:2,000

Secondary Antibodies Used:
• anti-Rabbit IgG HRP Conjugate: Promega (USA), Ref. No. 4018, Lot: 0000390794, Dilution:1:10,000
• anti-Mouse IgG HRP Conjugate: Promega (USA), Ref. No. W4021, Lot: 17331001, Dilution: 1: 10,000
• anti-Rat IgG HRP Conjugate: Sigma Aldrich,Cat. No: A9542-2mL, Lot: 019K4788, Dilution: 1:10,000

Validation

Primary Antibodies:
• anti-HA high affinity: see Fig. 1b, 2b, 3a, Suppl.Fig. 4a; https://www.sigmaaldrich.com/DE/en/product/roche/roahaha?gclid=EAIaIQobChMIkf-HtNvO8QIVz-J3Ch3tlQHMEAAYASAAEgIUevD_BwE
• anti-GST: see Fig. 1b, 1c, 2c, 4c; https://www.sigmaaldrich.com/DE/en/product/sigma/ge27457701
• anti-GFP: see Fig. 3b, 3d, 5a, 6b, 7c, 7d; https://www.thermofisher.com/antibody/product/A-11122.html?ef_id=EAIaIQobChMI9rbT2N7O8QIVA7p3Ch267gacEAAYASAAEgKlV_D_BwE:G:s&s_kwcid=AL!3652!3!459736943987!b!!g!!&gclid=EAIaIQobChMI9rbT2N7O8QIVA7p3Ch267gacEAAYASAAEgKlV_D_BwE
• anti-RFP: see Fig. 3b, 3d, 7d; https://www.chromotek.com/products/detail/product-detail/rfp-antibody-5f8/
• anti-RGS His: see Fig. 6a, 6b; https://www.qiagen.com/us/products/discovery-and-translational-research/protein-purification/tagged-protein-expression-purification-detection/anti-his-antibodies-bsa-free/
•anti-pS744: see Fig. 6a, 6b, 6c, 6d, 7c
•anti-NPH3: see Fig. 6a, 6c, 6d
Secondary Antibodies:
• anti-Rabbit : Promega (USA): https://www.promega.de/products/protein-detection/primary-and-secondary-antibodies/anti-rabbit-igg-h-and-l-hrp-conjugate/?catNum=W4011
• anti-Mouse: Promega (USA): https://www.promega.de/products/protein-detection/primary-and-secondary-antibodies/anti_mouse-igg-h-and-l-hrp-conjugate/?catNum=W4021
• anti-Rat:https://www.sigmaaldrich.com/DE/de/search/a9542
focus=products&page=1&perPage=30&sort=relevance&term=A9542&type=product
